# Nuclear receptor co-repressor NCOR2 and its relation to GPER with prognostic impact in ovarian cancer

**DOI:** 10.1007/s00432-023-04708-z

**Published:** 2023-05-02

**Authors:** Juliane Reichenbach, Patricia Fraungruber, Doris Mayr, Christina Buschmann, Fabian B. T. Kraus, Nicole Elisabeth Topalov, Anca Chelariu-Raicu, Thomas Kolben, Alexander Burges, Sven Mahner, Mirjana Kessler, Udo Jeschke, Bastian Czogalla, Fabian Trillsch

**Affiliations:** 1 Department of Obstetrics and Gynecology, University Hospital, Ludwig-Maximilian-University of Munich, Marchioninistr. 15, 81377 Munich, Germany; 2 Department of Obstetrics and Gynecology, University Hospital Augsburg, Augsburg, Germany; 3 Department of Pathology, Ludwig-Maximilian-University of Munich, Thalkirchner Strasse 36, 80337 Munich, Germany

**Keywords:** Ovarian cancer, Nuclear co-repressor 2, G-protein-coupled estrogen receptor, Immunohistochemistry, Epigenetic regulation, Chromatin remodeling

## Abstract

**Purpose:**

The significance of the non-classical G-protein-coupled estrogen receptor (GPER) as positive or negative prognostic factor for ovarian cancer patients remains still controversial. Recent results indicate that an imbalance of both co-factors and co-repressors of nuclear receptors regulates ovarian carcinogenesis by altering the transcriptional activity through chromatin remodeling. The present study aims to investigate whether the expression of the nuclear co-repressor NCOR2 plays a role in GPER signaling which thereby could positively impact overall survival rates of ovarian cancer patients.

**Methods:**

NCOR2 expression was evaluated by immunohistochemistry in a cohort of 156 epithelial ovarian cancer (EOC) tumor samples and correlated with GPER expression. The correlation and differences in clinical and histopathological variables as well as their effect on prognosis were analyzed by Spearman’s correlation, Kruskal–Wallis test and Kaplan–Meier estimates.

**Results:**

Histologic subtypes were associated with different NCOR2 expression patterns. More specifically, serous and mucinous EOC demonstrated a higher NCOR2 expression (*P* = 0.008). In addition, high nuclear NCOR2 expression correlated significantly with high GPER expression (cc = 0.245, *P* = 0.008). A combined evaluation of both high NCOR2 (IRS > 6) and high GPER (IRS > 8) expression revealed an association of a significantly improved overall survival (median OS 50.9 versus 105.1 months, *P* = 0.048).

**Conclusion:**

Our results support the hypothesis that nuclear co-repressors such as NCOR2 may influence the transcription of target genes in EOC such as GPER. Understanding the role of nuclear co-repressors on signaling pathways will allow a better understanding of the factors involved in prognosis and clinical outcome of EOC patients.

## Introduction

Epithelial ovarian cancer (EOC) remains the most life-threatening gynecological malignancies with an estimated 5-year survival of less than 45% and only 29% for advanced stage disease (Siegel et al. 2022). Initially, around 70% of the patients are primarily diagnosed at an advanced staged disease with dissemination to the entire abdominal cavity after presenting with unspecific symptoms and with the lack of reliable biomarkers (Barnholtz-Sloan et al. 2003). Due to molecular characteristics and differences in histogenesis and oncologic signaling pathways, EOC forms a heterogenous group of cancers consisting of five histological subtypes (Duska and Kohn 2017) which are major factors influencing clinical and biological behavior with impact on therapy and prognosis (Lalwani et al. 2011).

Signaling pathways important for cell growth, metabolism and inflammation are mediated by nuclear receptors, that are regulated through transcriptional co-regulatory proteins including co-activators and co-repressors (Mangelsdorf et al. 1995). The homeostatic balance between repressing and activating co-factors is key to regulate cell development. In contrast, dysregulation of these processes can promote either pro- or even anti-tumorigenic effects (Wong et al. 2014). NCOR2 is a nuclear co-repressor that was firstly known for its transcriptional silencing role of retinoid and thyroid hormone receptors and is referred to as SMRT (silencing mediator for retinoid and thyroid hormone receptors) (Hussein-Fikret and Fuller 2005; Sasaki et al. 2008). Appearing in large protein complexes up to 1.6–2 MDa, NCOR2 is forming the core, supported by protein components such as histone deacetylase 3 (HDAC3), transducin β-like protein 1 (TBL1) or TBL1-related protein 1 (TBLR1) and G-protein pathway suppressor 2 (GPS2) (Li et al. 2000). By chromatin remodeling, the co-repressor complex reduces transcriptional activity and consequently regulates different epigenetic cellular processes (Jepsen and Rosenfeld 2002). In malignant cells, the co-repressor machinery inhibits the normal transcriptional cycle under contribution of histone modifying enzymes and the DNA methylation machinery and leads to reduced transcriptional plasticity and consequently to an altered gene expression (Battaglia et al. 2010). Accordingly, NCOR2 expression was found in more than 70% of ovarian cancers (Havrilesky et al. 2001).

Several estrogen receptor isoforms such as ERα, ERβ and the membrane receptor GPER regulate ovarian cell differentiation and follicle and oocyte development and maturation (Bai et al. 2000). While especially serous and clear cell ovarian carcinomas derive from the fallopian tube and endometrium and not directly from the ovarian surface, the classical nuclear estrogen receptors α (ERα) and β (ERβ), demonstrated only minor effect for therapeutic approaches in EOC (Langdon et al. 2020). Non-genomic signaling on the other hand, is induced by binding of the non-classical G-protein-coupled estrogen receptor (GPER) (Filardo and Thomas 2012). G-protein-coupled receptors represent a large family of transmembrane molecules that mediate rapid intracellular responses to their extracellular ligands via cAMP or phosphatidylinositol signal transduction pathways and serve as potential drug targets (Hauser et al. 2018). Promoting estrogen-dependent physiological and pathophysiological processes, GPER expression was found in various cancer cell lines of reproductive tissues such as breast, endometrial, testicular and ovarian cancer (Prossnitz and Barton 2011; Pavlik et al. 2011; Revankar et al. 2005; He et al. 2009; Chevalier et al. 2012; Fujiwara et al. 2012). While the exact intracellular localization of GPER remains unclear, cytoplasmatic and nuclear occurrence were described (Otto et al. 2008; Zhu et al. 2018). Interestingly, foremost nuclear GPER expression served as significant independent negative prognostic factor for overall survival in EOC patients (Zhu et al. 2018).

The conflicting findings for GPER in EOC suggest that the complex regulation of transcriptional activity might be involved in both the pathogenesis and prognosis of EOC. Regulation by nuclear co-factors and co-repressors possibly alternates clinical outcome. The current study aims to explore a putative involvement of the nuclear co-repressor NCOR2 on the regulation of the gene GPER. A comprehensive understanding of the co-repressors’ role in EOC and their interaction with transcription factors and the target genes will allow a better understanding of the factors involved in prognosis and clinical outcome.

## Methods

### Patients and specimens

For the present study, 156 tumor samples with the clinical information, respectively, of ovarian cancer patients who underwent oncological surgery at the Department of Obstetrics and Gynecology, Ludwig-Maximillian’s-University Munich from 1990 to 2002 were collected, formalin fixated and paraffin embedded (FFPE). Additionally, the Munich Cancer Registry provided data about follow-up. All tumor samples included in this study originated from patients with malignant, non-borderline tumors and were further classified by a pathologist according to the histological subtypes serous (*n* = 110), endometrioid (*n* = 21), clear cell (*n* = 12), mucinous (*n* = 13). By WHO classification, serous ovarian cancer samples were defined as low- versus high-grade cancer, while endometrioid and mucinous subtypes as low- (G1), intermediate- (G2) and high-grade (G3) cancer. Clear cell ovarian cancer samples were always classified as high-grade cancer (G3) (Duska and Kohn 2017).

Of each EOC patient, three tissue specimen were obtained from the paraffin-embedded and formalin-fixated tumor blocks and compiled in tissue microarrays (TMA) paraffin blocks. By supervision of a pathologist, representative tumor sections of 2 μm were cut and aligned onto slides.

In the same sample set of 156 patients, various biomarkers for risk and prognostic assessment were already assessed in previous studies and the obtained data was hereby utilized to perform further analyses (Czogalla et al. 2019; Heublein et al. 2013).

### Immunohistochemistry

For NCOR2 staining of the FFPE microarrays of ovarian cancer samples, immunohistochemical procedures were conducted as previously described by our laboratory (Heidegger et al. 2017). Anti-NCOR2 IgG (Abcam, Cambridge, United Kingdom) served as primary antibody and was detected by polymer method (ZytoChem Plus HRP Polymer System mouse/rabbit, Zytomed Systems Berlin, Germany). The staining of FFPE ovarian cancer tissue samples was previously conducted by our lab incubating the sections with rabbit GPER IgG (BioGenex, Fremont, USA) (Heublein et al. 2012). Visualization was performed using chromogen diaminobenzidine (Dako, Hamburg, Germany). Counterstaining was performed using hematoxylin (Waldeck-Chroma, Münster, Germany). At all steps, system controls were included.

The immunohistochemical staining reaction in the nuclei and cytoplasm of the tumor cells was assessed by a Leitz photomicroscope (Wetzlar, Germany) applying a semi-quantitative immunoreactivity scoring system (IR-score, Remmele’s score). Therefore, staining intensity (no staining = 0, weak staining = 1, moderate staining = 2, strong staining = 3) is defined in relation to the percentage of stained cells (less than 10% of positive cells = 1, 11–50% of positive cells = 2, 51–80% of positive cells = 3, more than 81% of positive cells = 4) (Remmele et al. 1986).

### Staining evaluation

A receiver operating characteristic curve (ROC) was utilized to generate reliable cut-off values for the IR-Score of the NCOR2 staining. Hereby, the true positive rate (corresponding to the sensitivity) is related in a plot to the false positive rate (calculated as 1 − specificity). The most error-free points, which are those with highest sensitivity and specificity are determined by Youden’s J statistic (Lasko et al. 2005). For NCOR2, samples with IRS ≤ 6 were considered to show low and samples with IRS > 6 to show high expressed according to a median IRS of 6 as cut-off point.

Staining results for GPER in the same tumor sample group that defined a median IRS of 8 as cut-off point for low (IRS ≤ 8) and high (IRS > 8) expression were already published by our laboratory and taken into consideration (Heublein et al. 2013).

### Statistical analysis

Statistical analysis was conducted with IBM SPSS Statistics Version 25.0 (PASW Statistic, SPSS Inc., IBM, IL, USA). Spearman analysis was carried out for correlation analysis of NCOR2 and GPER. Overall survival was calculated by Kaplan–Meier estimates (log-rank). *P*-values < 0.05 were considered as statistically significant.

## Results

### Clinical and pathological characteristics

Clinical and pathological characteristics of our ovarian cancer patient collective, such as data on histology, lymph node status, FIGO classification, age and information about follow-up, were already described and published previously by our group (Table 1) (Czogalla et al. 2019; Heublein et al. 2013).

**Table 1:** Clinicopathological data

Clinicopathological aspects	*N*	Percentage (%)
Histology		
Serous	104	70.50
Low grade	24	21.80
High grade	80	72.70
Clear cell	12	7.70
Endometrioid	21	13.50
Mucinous	13	8.30
Lymph node		
pNX	61	39.10
pN0	43	27.60
pN1	52	33.30
FIGO		
I	35	23.10
II	10	6.60
III	103	68.20
IV	3	2.00
Age		
≤ 60 years	83	53.20
> 60 years	73	46.80

Clinicopathological data about the ovarian cancer patient collective (Table modified according to Czogalla et al. 2019)

### NCOR2 expression

**Fig. 1:**
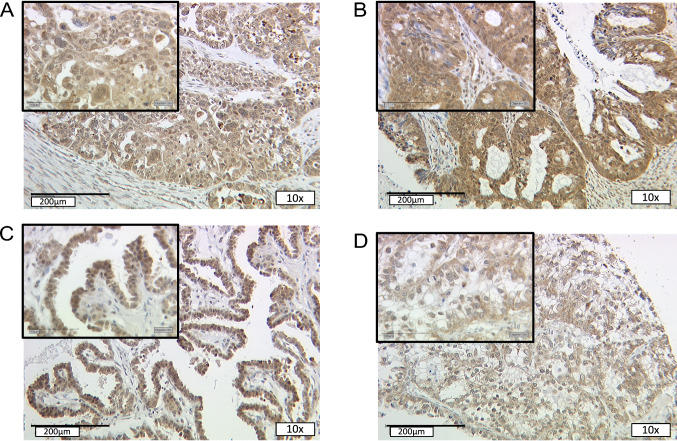
Detection of NCOR2 with immunohistochemistry. Nuclear NCOR2 staining in the subtypes serous (**A**), mucinous (**B**), endometrioid (**C**) and clear cell (**D**)

**Fig. 2:**
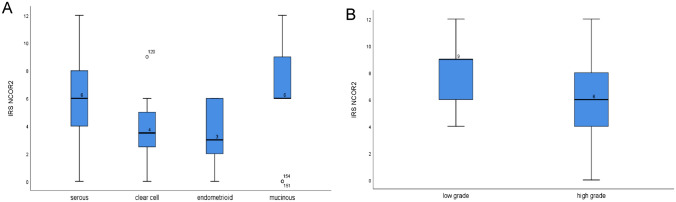
Boxplot graphs for NCOR2 IR-scores of the respective subtypes. Significantly different median IR-scores were found for the histological subtypes, respectively (*P* = 0.008), **A** for serous, clear cell, endometrioid and mucinous EOC and **B** for low-grade and high-grade serous EOC

For 123 of the evaluable 152 cases (82%), NCOR2 nuclear staining was conducted properly and median immunoreactivity with an IRS of 6.0 (SD = 3.1) was detected. In terms of histologic subtypes, significantly higher median IR-scores were found in serous (IRS = 6) and mucinous carcinomas (IRS = 6, compared to weaker expression in endometrioid EOC (IRS = 3, *P* = 0.008) (Figs. 1A–D, 2A). For serous EOC, different immunoreactivity was observed depending on grading, with weaker NCOR2 expression in high-grade (IRS = 6) compared to low-grade serous histology (IRS = 9, *P* = 0.002) (Fig. 2B). No further correlations with statistical significance were noted for NCOR2 with regard to other clinicopathological data as listed in Table 1.

### Correlation analysis for NCOR2 and GPER

Nuclear NCOR2 immunoreactivity was correlated with the previously reported expression of estrogen receptors. Here, a significant correlation of high NCOR2 expression with GPER immunoreactivity was noted (*P* = 0.008; cc = 0.245), while no significant correlation was observed for ERα and ERβ receptors (Table 2).

**Table 2:** Correlation analysis of NCOR2 with GPER, ERα and ERβ

			NCOR2	GPER	ERα	ERβ
Spearman’s rho	NCOR2	cc	1.000	0.247	0.071	0.173
		*p*	–	0.008	ns (0.446)	ns (0.064)
		*N*	–	115	117	115

Spearman correlation showed positive correlation of NCOR2 immunoreactivity with GPER expression, while no correlation was found with ERα and ERβ staining

### High NCOR and GPER expression is associated with improved overall survival

In Kaplan–Meier analysis to detect possible influences of the immunophenotypes on overall survival (OS), high NCOR2 expression was not associated with significant impact on prognosis (median OS 52.3 months for NCOR2 IRS > 6 versus 50.9 months for NCOR2 IRS < 6; *P* = 0.600) (Fig. 4A). Patients with a high GPER expression had a median OS improvement of 17.3 months in comparison to patients with low GPER expression (median OS 35.0 months for IRS < 8 versus median OS 52.3 months, for IRS > 8), however not statistically significant (*P* = 0.176) (Fig. 4B).

**Fig. 3:**
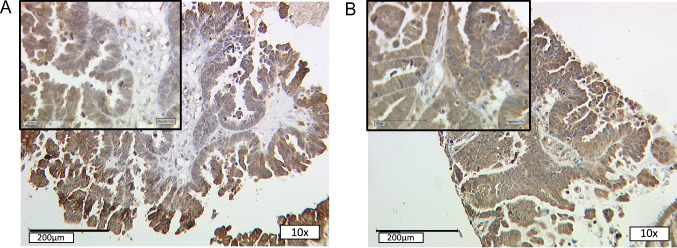
GPER (**A**) and NCOR2 (**B**) staining in the same individual. For the same patient with serous EOC high GPER and high NCOR2 staining was detected

**Fig. 4:**
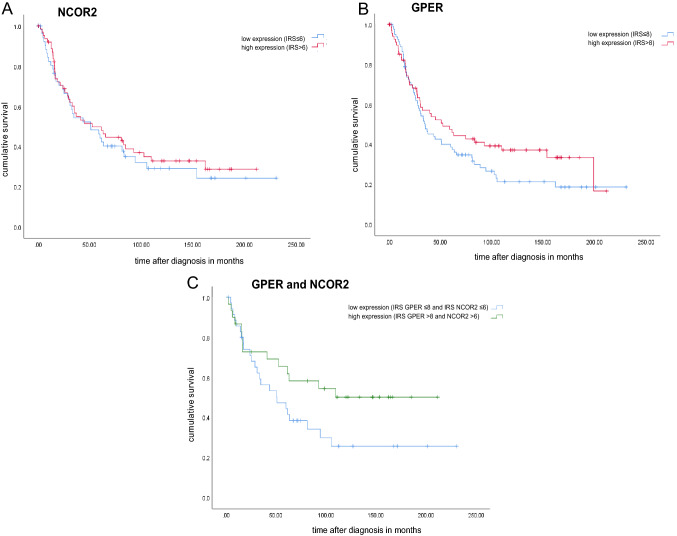
Kaplan–Meier estimates. Kaplan–Meier estimates of NCOR2 expression (**A**), GPER expression (**B**) and combined GPER and NCOR2 expression (**C**) were analyzed. Both NCOR2 and GPER expression were associated with prolonged overall survival (**A**, **B**), however, without statistical significance. A significant effect on overall survival was detected for the combined high GPER and NCOR2 expression (**C**)

Consistent with the hypothesized contribution of NCOR2 to GPER guided signaling pathways, the combination of high NCOR2 as well as GPER expression was associated with a significant effect on OS (*P* = 0.048). OS was significantly longer for patients with NCOR2 IRS > 6 and GPER IRS > 8 with a median OS of 105.1 months compared to 50.9 months with low expression of both markers (Figs. 3, 4C).

### Multivariate analysis

A multivariate Cox regression analysis was developed to evaluate the influence of the variables age, histological type, FIGO stage and expression of NCOR2 and GPER on OS. In this context, only FIGO stage was identified as a significant independent prognostic factor with a Hazard ratio of 1.733 (CI 1.113–2.698; *P* = 0.015) (Table 3).

**Table 3:** Multivariate analysis

Covariate	Coefficient	Hazard ratio	*P* value	95% CI
Age (< 60y vs. ≥ 60y)	0.223	1.250	0.395	0.748–2.089
Histology (HGSOC vs. LGSOC vs. clear cell vs. endometrioid vs. mucinous)	− 0.128	0.880	0.453	0.630–1.229
FIGO (I vs. II vs. III vs. IV)	0.550	1.733	0.015*	1.113–2.698
NCOR2 (low vs. high expression)	0.044	1.045	0.908	0.491–2.227
GPER and NCOR2 (expression of either GPER or NCOR2 vs. expression of both)	0.045	1.046	0.874	0.600–1.824

In the multivariate Cox regression analysis only the FIGO stage was detected as a significant independent prognostic factor as indicated by asterisks (*P < 0.05)

## Discussion

In the present study, we investigated the expression of the nuclear co-repressor NCOR2 in EOC and its potential role in estrogen-dependent physiological and pathophysiological processes promoted by the G-protein coupled estrogen receptor GPER through non-genomic signaling. Co-expression of both GPER and NCOR2 showed significant changes in clinical outcome in EOC patients, highlighting not only the molecular, but also the prognostic significance of the transcriptional regulation altered by NCOR2. In accordance to differences in the pathogenesis of EOC (Kurman and Shih 2011), the NCOR2 expression varied significantly accordingly to histological subtypes and was independent from other clinical–pathological data. Highest NCOR2 immunoreactivity was observed in serous carcinomas and was dependent on tumor grading. Since subtype-specific impact of NCOR2 expression on overall survival by Kaplan–Meier estimates was not statistically significant, the present study further evaluated general molecular mechanisms of ovarian cancer biology rather than focusing on subtype-specific analyses. Isolated evaluation of high NCOR2 co-repressor as well as high GPER expressing tumors could not reveal significant impact on OS, whereas evaluation of both factors together indicated that the tumors exhibiting the combination of both, high NCOR2 and high GPER expression, seem to have significantly improved OS with a median improvement of 54.2 months. Consequently, these results suggest an association with a possible regulating role of the nuclear co-repressor NCOR2 in GPER-mediated signaling, which merits further evaluation.

As a transcription co-regulator of metabolic processes, NCOR2 is involved in the development of various cancer entities by unbalancing pro- and anti-inflammatory signaling pathways (Mottis et al. 2013). For primary ovarian cancers, its expression was found in up to 70% of tumor tissues (Havrilesky et al. 2001). Structural investigations detected NCOR2 appearing in a large co-regulatory complex that assembles multiple and context-dependent partner proteins (Oberoi et al. 2011). The NCOR2 co-repressor complex disrupts transcription of various target genes through chromatin remodeling, leading to altered cellular processes and to malignancy through induced transcriptional arrest (Battaglia et al. 2010). Some of the complex’ proteins represent substantial core components as they account for the repressive function (Oberoi et al. 2011). Each of these components was shown to get involved in carcinogenesis. Transducing β-like related 1 (TBLR1), for instance, is taking an ambiguous role, having either co-repressing or co-activating function depending on the affected cell type and interacting hormone receptor (Wu et al. 2016). It appeared to be significantly upregulated in ovarian cancer cells and served as predictor for the clinical outcome of EOC patients (Havrilesky et al. 2001; Wu et al. 2016; Ma and Yu 2017). Showing a significantly higher expression in both the nucleus and cytoplasm of breast and ovarian cancer cells compared to benign tissues, it activates cell proliferation and accelerates migration and invasion through ER-independent and ER-dependent pathways (Wu et al. 2016). However, in EOC, granulosa cell tumors of the ovary and healthy ovarian tissue, no significant correlation of NCOR1 and NCOR2 with ERβ was found, consistent with our results of NCOR2 expression and their correlation to ERα/ERβ (Hussein-Fikret and Fuller 2005).

Depending on the hormonal signaling context, the NCOR2 complex gets involved into the transcriptional activity of different nuclear receptors, including steroid hormone receptors such as estrogen, androgen and progesterone receptors and is incorporated in different regulation processes (Jepsen and Rosenfeld 2002; Wong et al. 2014). Various studies affirm that an appropriate suppression of NCOR2 expression is a key aspect for proper cell signaling. In contrast, aberrant function can promote cancer and disease progression. In breast cancer cells, NCOR2 was proposed as a candidate to initiate cancer cell growth by modifying the transcriptional activity of ERα or directly influencing the ERα expression (Ciriello et al. 2013; Dobrzycka 2003; Kurebayashi et al. 2000). In prostate cancer, loss of the repressive function of NCOR2 altered the AR response to ligands and contributed to cancer development (Godoy et al. 2012). By interacting with other co-repressing proteins, NCOR2 was identified as a fundamental modulator of the estrogen receptor in breast cancer patients treated with tamoxifen as adjuvant antihormonal therapy. In these, aberrant NCOR2 appeared to modify the hormone receptor response to tamoxifen while low NCOR2 levels predicted drug resistance against tamoxifen (Gong et al. 2018). For serous ovarian cancer patients, NCOR2 was identified as a possible predictive biomarker of chemotherapy response among seven other genes in a gene array study, correlating with resistance to chemotherapy (Fekete et al. 2020). By epigenetic regulation, co-repressors play a context-dependent role in biological processes and deregulated function might unbalance homeostasis and therefore accelerate malignant degeneration (Mottis et al. 2013).

Estrogen-dependent signaling regulated by GPER was found in several tumor entities (Chevalier et al. 2012; Fujiwara et al. 2012; He et al. 2009; Prossnitz and Barton 2011; Revankar et al. 2005). Thereby, the role of GPER expression in ovarian cancer tumorigenesis either as promoting or as suppressing factor remains still subject to current research: for either Erα-negative or Erα-positive ovarian cancer cells, GPER-mediated signaling pathways were found to promote ovarian cancer cell proliferation (Liu et al. 2014; Albanito et al. 2008). In contrast, GPER expression might inhibit cell growth and therefore serve as tumor suppressor and as a positive prognostic factor for disease-free survival (Ignatov et al. 2013). While comparable nuclear and cytoplasmatic occurrence was described, the specific intracellular localization and signaling pathway has not been identified yet. On a differentiated analysis of a previous study, only nuclear GPER expression was identified as an independent negative prognostic factor (Zhu et al. 2018). The study assumed that nuclear rather than cytoplasmatic occurrence drives carcinogenesis and therefore leads to impaired outcome (Zhu et al. 2018). Further investigation with a discrimination of nuclear and cytoplasmatic expression of GPER in EOC might serve as a future approach to explain these contradictory findings.

However and in accordance with our results, effects on overall survival are not depending on GPER expression alone. The present results indicate a possible regulating role of the NCOR2 nuclear co-repressor complex on GPER-regulated pathways in EOC. High expression of both immunophenotypes was shown to be significantly correlated with a positive impact on OS in ovarian cancer patients possibly indicating a role to serve as a reliable prognostic marker. Approximately 34% of drugs approved by the American Food and Drug Administration (FDA) exert their therapeutic effects by targeting G-protein-coupled receptors (Hauser et al. 2018). Our new insights to the previously relatively unknown role of GPER in EOC carcinogenesis make GPER a potential novel target for treatment strategies. Furthermore and more importantly, drugs with epigenetic targets such as Small inhibiting molecules, Histone deacetylase inhibitors and demethylation agents might be able to modulate the function of the NCOR2 complex and might therefore alter response to chemotherapy and affect the clinical outcome of EOC patients (Graham et al. 2009).

Based on the results of the present study, we hypothesize that NCOR2 mediated modulation of nuclear GPER-mediated signaling pathways is responsible for the improved prognosis. Some limitations of our study should be taken into consideration for the interpretation and evaluation of the present data. Since EOC comprise a heterogenous group of several histological subtypes that differ in biological, clinical and prognostic behavior, our study is limited by a relatively small sample subset that allows rather general than subtype-specific conclusions regarding the ovarian cancer biology. As a retrospective dataset, additional data on patient characteristics are lacking for a deeper exploration considering factors such as menopausal status, estrogen levels and exposure to hormonal replacement therapy. Moreover, further studies and experimental methods will be necessary to elucidate not only the static receptor expression by immunohistochemistry, but also mechanisms regarding the regulation of gene transcription, modulation of receptor dynamics and ultimately its exact biological function. Based on the presented results, these consecutive experiments are already in preparation by our group and will be prospectively followed. Accordingly, the current study might serve as a potential starting point to further explore the complex molecular implications of the epigenetic regulator NCOR2 and its components on processes involved in ovarian cancer development, such as GPER-mediated signaling pathways. A comprehensive understanding of the mechanisms of chromatin regulation and transcription activity in ovarian cancer development enables future patient-specific therapeutic approaches.

## Data Availability

The datasets generated during and analyzed during the current study are available from the corresponding author on reasonable request.

## Abbreviations

cAMP: Cyclic adenosine monophosphate
cc: Correlation coefficient
CI: Confidence interval
EOC: Epithelial ovarian cancer
ERα: Estrogen receptor α
ERβ: Estrogen receptor β
FDA: Food and Drug Administration
FFPE: Formalin fixated and paraffin embedded
FIGO: Fédération Internationale de Gynécologie et d'Obstétrique
GPER: G-protein-coupled estrogen receptor
GPS2: G-protein pathway suppressor 2
HDAC3: Histone deacetylase 3
HGSOC: High-grade serous ovarian cancer
IgG: Immunoglobulin G
IRS: Immunoreactive Score
LGSOC: Low grade serous ovarian cancer
MDa: Megadalton
*N*: Number of patients
NCOR2: Nuclear co-repressor 2
ns: No significance
OS: Overall survival
*P*: Two-tailed significance
ROC: Receiver operating characteristic
SD: Standard deviation
TBL1: Transducin b-like protein 1
TBLR1: TBL1-related protein 1
TMA: Tissue micorarrays
vs.: Versus
WHO: World Health Organization
